# Supplementary material for: Impulsivity and body fat accumulation are linked to cortical and subcortical brain volumes among adolescents and adults
Source: Sci Rep. 2019 Feb 22;9:2580. doi: 10.1038/s41598-019-38846-7 (PMC6385240; doi:10.1038/s41598-019-38846-7)
Supplement: Supplementary file 1 — Supplementary Info [file 41598_2019_38846_MOESM1_ESM.docx]

**Supplementary Information:**

**Impulsivity and body fat accumulation are linked to cortical and subcortical brain volumes among adolescents and adults**

Naomi Kakoschke, Valentina Lorenzetti, Karen Caeyenberghs, Antonio Verdejo-García

**Supplementary Results**

**Associations between BMI and impulsivity**

We found negative associations between BMI and negative urgency (*r* = -.263, *p* =. 005), sensation seeking (*r* = -.350, *p* <.001), and premeditation (*r* = -.243, *p* =. 009). No other associations between BMI and impulsivity survived significance.

**Associations between BMI, impulsivity and regional brain volumes**

We found that BMI was positively associated with the volumes of the NAcc (left: *r* = .239, *p* = .007), amygdala (left: *r* = .249, *p* = .005, right: *r* = .243, *p* =. 006), and cerebellum white matter (left: *r* = .322, *p* <.001, right: *r* = .242, *p* =. 006). No other associations between % body fat and impulsivity with regional brain volumes survived significance.

**Associations between BMI, impulsivity and regional brain volumes by age group**

Partial correlational analyses between BMI and impulsivity with regional brain volumes were also conducted separately for adults and adolescents. We found a positive association between BMI and left frontal pole volume in adolescents (*r* = .354, *p* = .005), but not adults (*r* = -.099, *p* = .449).
